# Mouse Natural Killer (NK) Cells Express the Nerve Growth Factor Receptor TrkA, which Is Dynamically Regulated

**DOI:** 10.1371/journal.pone.0015053

**Published:** 2010-12-01

**Authors:** Natacha Ralainirina, Nicolaas H. C. Brons, Wim Ammerlaan, Céline Hoffmann, François Hentges, Jacques Zimmer

**Affiliations:** 1 Laboratory of Immunogenetics and Allergology, Centre de Recherche Public de la Santé (CRP-Santé), Luxembourg, Luxembourg; 2 Core Facility Flow Cytometry, Centre de Recherche Public de la Santé (CRP-Santé), Luxembourg, Luxembourg; 3 Laboratory of Plant Molecular Biology, Centre de Recherche Public de la Santé (CRP-Santé), Luxembourg, Luxembourg; Chinese University of Hong Kong, Hong Kong

## Abstract

**Background:**

Nerve growth factor (NGF) is a neurotrophin crucial for the development and survival of neurons. It also acts on cells of the immune system which express the NGF receptors TrkA and p75^NTR^ and can be produced by them. However, mouse NK cells have not yet been studied in this context.

**Methodology/Principal Findings:**

We used cell culture, flow cytometry, confocal microscopy and ELISA assays to investigate the expression of NGF receptors by NK cells and their secretion of NGF. We show that resting NK cells express TrkA and that the expression is different on NK cell subpopulations defined by the relative presence of CD27 and CD11b. Expression of TrkA is dramatically increased in IL-2-activated NK cells. The p75^NTR^ is expressed only on a very low percentage of NK cells. Functionally, NGF moderately inhibits NK cell degranulation, but does not influence proliferation or cytokine production. NK cells do not produce NGF.

**Conclusions/Significance:**

We demonstrate for the first time that mouse NK cells express the NGF receptor TrkA and that this expression is dynamically regulated.

## Introduction

Nerve growth factor (NGF) is a neurotrophin crucial for the development and survival of neurons [1]. It is known that this molecule acts also on cells of the immune system and can be produced by such cells, in particular eosinophils, monocytes/macrophages, granulocytes, mast cells as well as B and T lymphocytes [2], [3]. NGF is an autocrine growth and survival factor for B cells [2], [3]. It can also influence proliferation of T lymphocytes [2], [3]. NGF acts through two types of receptors: (i) a high affinity receptor which is the tyrosine kinase TrkA, specific for NGF, and (ii) a low affinity receptor called p75^NTR^, which is a pan-neurotrophin receptor recognizing all neurotrophins of the NGF family (NGF, brain-derived neurotrophic factor, neurotrophin-3 and neurotrophin-4) [1]–[3]. Both types of receptors have been found on immune cells, with TrkA-transmitted signals usually being anti-apoptotic and stimulating [1]–[3], whereas p75^NTR^ rather transmits pro-apoptotic signals [2].

Despite intense investigations on NGF and its receptors within the immune system, studies on NGF in the context of natural killer (NK) cells have, to the best of our knowledge, never been performed until very recently [4]. NK cells are lymphocytes different from B and T cells. They are capable of killing tumor cells and virally infected cells without prior immunization or activation (although their functional properties are enhanced after cytokine-mediated stimulation) and are thus part of the innate immune response. Due to their abundant cytokine production, they also influence adaptive immunity [5], [6]. In addition, the existence of regulatory [6] and memory [5] NK cells has been recently demonstrated.

NK cell functions are tightly regulated by a balance between the messages transmitted by activating receptors and those transmitted through inhibitory receptors. Most of the latter recognize classical or non classical major histocompatibility complex (MHC) class I molecules on surrounding cells. In the mouse, they are represented by NKG2A and by the Ly49 family (both are members of the C-type lectin superfamily). When MHC class I molecules are down-regulated or absent, which frequently occurs during tumor transformation or viral infections, the diseased cells are selectively killed by the NK cells whereas normal cells are spared (self tolerance of NK cells) [6], [7]. However, killing and cytokine production can only be performed by “licensed” or “educated” NK cells characterized by the expression of at least one inhibitory receptor for self MHC class I molecules [8], [9]. NK cells without such receptor(s) are maintained within the immune system, but they are “unlicensed”, which means that they are hypo-responsive to stimulation through their activating receptors [8]. In contrast, they respond more efficiently to viruses than the “educated” cells, at least in the context of cytomegalovirus infection [10].

In this paper, we investigated if NGF is produced by NK cells and if NK cells express NGF receptors. We show for the first time that normal mouse NK cells can express TrkA and that this receptor is dynamically regulated on NK cells. In addition, we performed functional NK cell studies which revealed a tendency of NGF to negatively influence NK cell degranulation. NK cells do not produce NGF.

## Materials and Methods

### Mice

C57BL/6 (B6) mice were purchased from Harlan (Horst, The Netherlands). They were housed in a specific pathogen free animal facility with a light/dark cycle of 12 hours and had unlimited access to irradiated food and sterilized water. Mice were used between 6 and 12 weeks of age. All animal experiments were performed in accordance with the current European directives and Luxemburgish laws. The latter do not require presenting projects that involve animal models to an Ethics Committee.

### NK cell purification, expansion and activation

Spleens were aseptically removed and single cell suspensions were prepared after dissociation of the spleen through a cell strainer. Red blood cells were lysed by incubation in ACK buffer (Lonza, Basel, Switzerland) for 1 minute. This was followed by two washes in Dulbecco's modified Eagle's Medium (DMEM) with 10% FCS, penicillin/streptomycin, 10 mM HEPES buffer and 50 µM 2-ME (complete medium). All cell culture ingredients were purchased from Invitrogen (Paisley, UK). The splenocytes were pre-purified over a nylon wool column (G. Kisker GbR, Steinfurt, Germany) in order to remove the majority of B cells. Enriched NK and T cells (nylon wool non adherent splenocytes, NWNA) were then either put immediately in culture or were further purified with the NK cell isolation kit from Miltenyi Biotec (Bergisch-Gladbach, Germany) according to the manufacturer's instructions.

NWNA splenocytes or purified NK cells were cultured at a density of 2×10^6^ cells/ml in complete medium in cell culture plates for various periods of time. Cultures were supplemented with recombinant human interleukin 2 (IL-2, R & D Systems, Abingdon, UK) at 1000 U/ml with or without recombinant mouse NGF (R & D Systems) at 100 ng/ml. At the end of the culture period, adherent NK cells were harvested with EDTA (Sigma, Bornem, Belgium). Viability of the cells was assessed with the Trypan Blue dye exclusion test.

### Flow cytometry

Splenocytes or purified NK cells were first incubated for 5 minutes with anti-CD16/CD32 antibody (Ab; 1 µg/10^6^ cells) and 5% goat serum for TrkA or donkey serum for p75^NTR^ stainings, to reduce non specific binding. Then the TrkA (polyclonal rabbit recognizing human, mouse and rat TrkA, Santa Cruz Biotechnology, Santa Cruz, CA) and p75^NTR^ (polyclonal goat, R & D Systems) Ab were added for 30 minutes at 4°C. Normal rabbit IgG and normal goat IgG were used as negative controls. After two washing steps in FACS buffer, cells were incubated again for 30 minutes, at 4°C in the dark, in the presence of fluorochrome-conjugated Ab directed against various surface markers, as well as goat anti-rabbit IgG/PE and donkey anti-goat IgG/PE (both from Jackson Immunoresearch, Soham, Cambridgeshire, UK), respectively. After additional washing steps, cells were fixed with Cellfix (BD Biosciences, Erembodegem, Belgium). Samples were analyzed on a FACSCanto flow cytometer with FACSDiva software (BD Biosciences) and FlowJo (Treestar, Ashland, OR).

The phenotype of the NK cells after culture with or without NGF was analyzed by flow cytometry after direct staining of the splenocytes with the following fluorochrome-conjugated monoclonal anti-mouse Ab: Ly49C/I-FITC, Ly49D-FITC, Ly49G2-FITC, Ly49A-PE, Ly49F-PE, NKG2D-PE (BD Biosciences), Ly49H-FITC, CD11a-FITC, CD18-FITC, CD27-FITC, CD54-FITC, NKG2A/C/E-FITC, CD94-PE, CD3-PECy7, NK1.1-PECy7, CD11b-APC, DX5-APC, NK1.1-APC, CD3-Alexa Fluor 780 (eBioscience, San Diego, CA), NK1.1-Alexa Fluor 780, CD69-PE (Biolegend, San Diego, CA).

Apoptosis assays were performed with the annexin V/propidium iodide (PI) test. Cells were cultured with IL-2 or IL-2+ NGF for 3 days. Then the cells were incubated in a binding buffer (10 mM HEPES/NaOH pH 7.4, 140 mM NaCl, 2.5 mM CaCl_2_) and stained with Ab against NK1.1 (FITC) and CD3 (Alexa Fluor 780) as well as with annexin V-APC for 30 minutes at room temperature. Cells were washed and resuspended in binding buffer. Immediately before running the samples on the flow cytometer, PI was added.

### Confocal microscopy

Cells were allowed to settle onto poly-L-lysine (Sigma) coated coverslips for 15 minutes. Cells were then fixed with 4% paraformaldehyde (Sigma) and incubated with 0.1 M glycin for 10 minutes. After a washing step, cells were permeabilized by incubation with 1% BSA/PBS/saponin for 15 minutes and labeled with the rabbit anti-mouse TrkA or goat anti-mouse p75^NTR^ Ab for 30 minutes. The detection Ab used were anti-rabbit IgG-Alexa Fluor 488 and anti-goat IgG Alexa Fluor 488, respectively (incubation of 30 minutes in the dark). Finally cells were washed with 1% BSA/PBS/saponin. The nuclear marker DAPI was added for 10 minutes. Samples were mounted between slide and coverslip in gel mounting medium (Sigma) and analyzed on a confocal microscope LSM 510 META (Zeiss, Jena, Germany) equipped with a 63X Plan-Apochromat oil immersion objective (numerical aperture 1.4). Alexa-488 and DAPI were detected by exciting samples at a wavelength of 488 nm and 405 nm respectively, and using a 505–530 nm (for green) or a 420–480 nm (for blue) band-pass emission filter.

### Proliferation assay

Splenocytes or purified NK cells were labeled with 5(6)-carboxyfluorescein diacetate N-succinimidyl ester (CFSE, Sigma) at 10 µM for 15 minutes at 37°C and washed twice in complete medium. Then labeled cells were cultured in complete medium with IL-2 (1000 U/ml) alone or with IL-2 and recombinant mouse NGF (rmNGF, concentrations from 0–10^4^ pm) for three days.

### CD107a degranulation assay

96 well plates were coated overnight with the purified anti-NK1.1 Ab PK136 (Immunotools, Friesoyhte, Germany) at 4°C. The day after, supernatant was removed from the plates and NWNA splenocytes were incubated during 4 hours at 37°C in complete medium under the following conditions: (i) no stimulation, (ii) IL-2 alone (1000 U/ml), (iii) NGF alone (100 ng/ml), (iv) IL-2+ IL-12 (10 ng/ml) + IL-18 (20 ng/ml), and finally (v) IL-2+ IL-12+ IL-18+ NGF. A FITC-conjugated anti-CD107a Ab as well as Golgi Plug (BD Biosciences) were present during the incubation time. After the 4 hours, cells were stained with CD3-PECy7 and DX5-APC Ab and analyzed by flow cytometry. The experiment was performed similarly on NK cells activated during three days in the presence of IL-2.

### Intracellular cytokine staining

NWNA splenocytes were cultured with IL-2 alone or with IL-2+ NGF during 3 days at 37°C. On day 2, cells were stimulated with IL-2+ IL-12+ IL-18 with or without NGF overnight. The following morning, GolgiPlug (BD Biosciences) was added to the cells for additional 4 hours of incubation. After this period, cells were surface-stained with CD3-PECy7 and NK1.1-APC Ab for 30 minutes at 4°C in the dark. After fixation and permeabilization with Cytofix/Cytoperm solution (BD Biosciences) during 15 minutes, cells were stained with IFNγ/PE Ab (ebioscience) for 30 minutes at 4°C in the dark, washed twice in PermWash solution and analyzed by flow cytometry. The same experiment was also performed with fresh NK cells kept overnight at 4°C.

### NGF ELISA assay

For the determination of NGF secretion, purified splenic NK cells were cultured in 96 well plates at a density of 2×10^6^ cells/ml. After 4 days, cell-free supernatants were harvested and frozen until use. NGF levels in the supernatant were determined by the NGF ELISA kit from Chemicon International (Hofheim, Germany) according to the manufacturer's instructions. The detection limit of NGF was 10–15 pg/ml.

### Statistical analysis

Results are expressed as means ± SEM from independent experiments. Variance ANOVA one way Newman-Keuls test was used for data analysis with GraphPad Prism software.

## Results

### NK cells express TrkA

**Figure 1:**
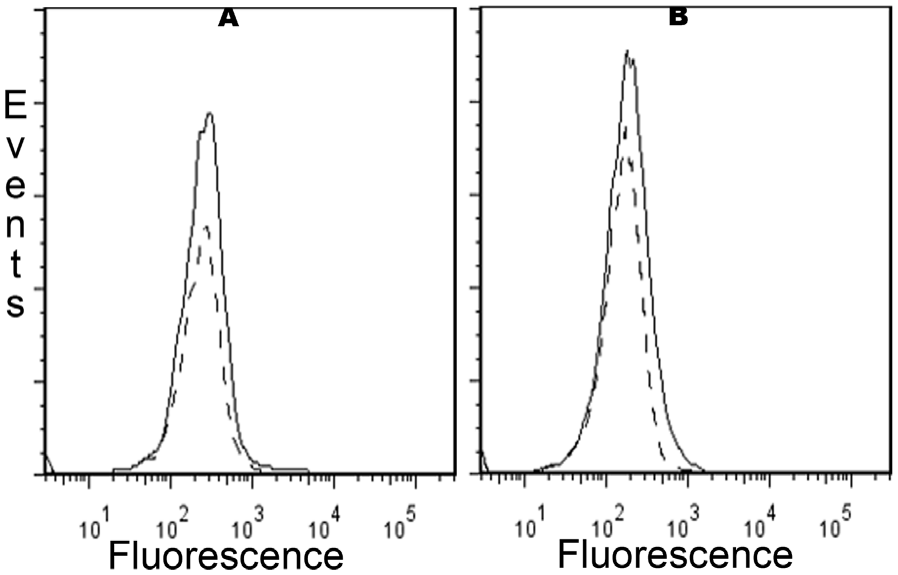
Low expression of the pan neurotrophin receptor p75^NTR^ on fresh and activated mouse NK cells. Splenocytes were stained with anti-NK1.1, anti-CD3 and anti-p75^NTR^ Ab or isotype control as described in Materials and Methods, and analyzed by flow cytometry. Dead cells were excluded by staining with Live Dead cell marker. A gate was set on NK cells (CD3-NK1.1+). Only a weak expression of p75^NTR^ (grey lines) relative to isotype control (dashed lines) was observed at day 0 (left panel) and after 5 days of culture in the presence of IL-2 (right panel). Data shown are from one representative experiment out of three performed.

**Figure 2:**
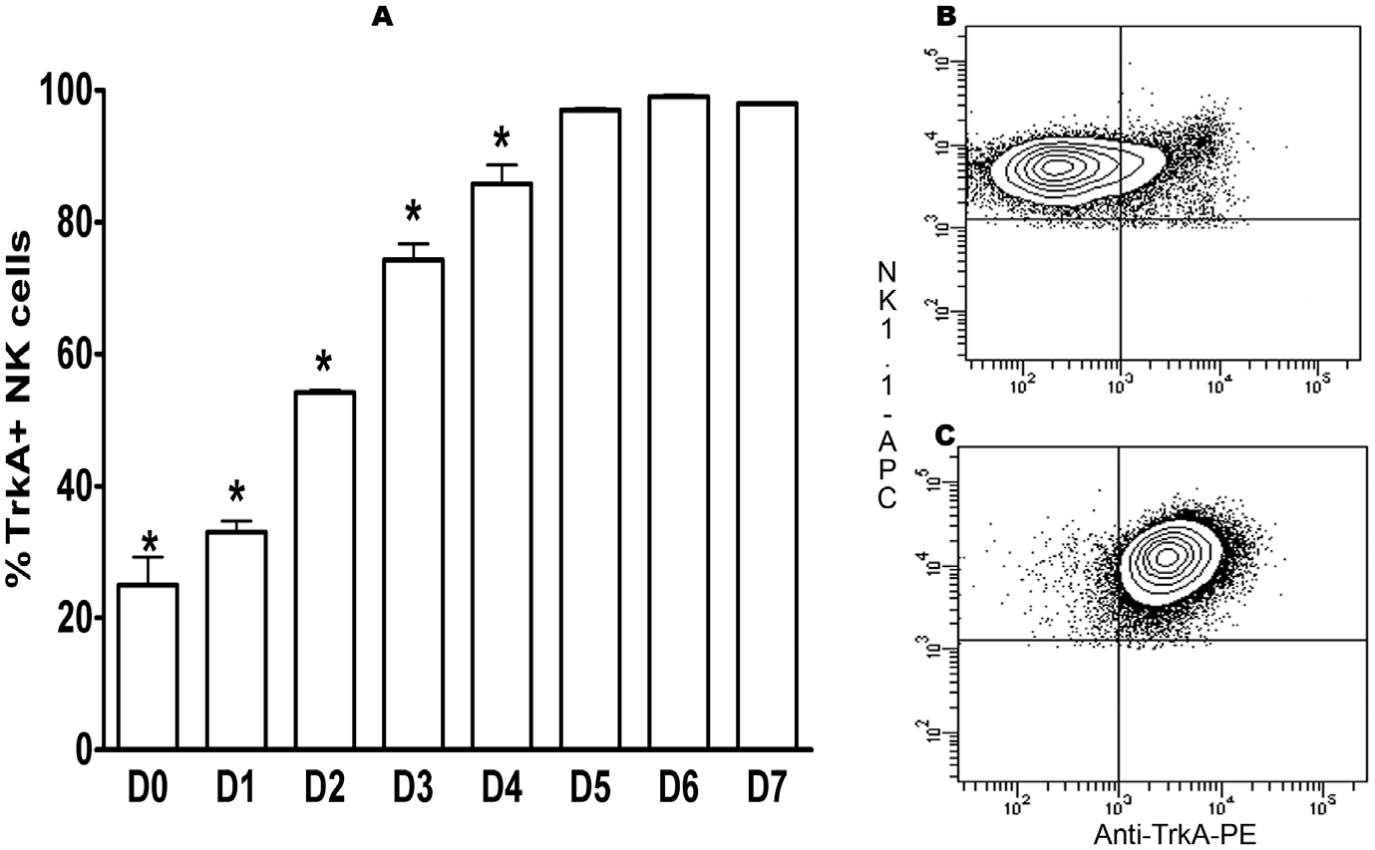
Kinetics of the expression of the high affinity NGF receptor TrkA on mouse NK cells. Splenocytes were stained with anti-NK1.1, anti-CD3 and anti-TrkA Ab or isotype control as described in Materials and Methods, and analyzed by flow cytometry. Dead cells were excluded by staining with Live Dead cell marker. A gate was set on NK cells (CD3-NK1.1+), and the percentage of TrkA+ NK cells was determined. A: kinetics of TrkA expression from day 0 to day 7 (culture in the presence of IL-2). The data shown are the means ± SEM of three experiments. *: p<0.05: B: TrkA expression on fresh splenic NK cells represented as contour plot. Data shown are from one representative experiment out of three performed. C: TrkA expression on day 5 of culture with IL-2 represented as contour plot. Data shown are from one representative experiment out of three performed.

We investigated by flow cytometry the expression of NGF receptors on NK cells present within fresh splenocytes from B6 mice. Whereas p75^NTR^ could be detected only on a very low number of NK cells (Fig. 1), TrkA was expressed by approximately 20% of the cells (Fig. 2A, Fig. 2B). The presence of TrkA in purified spleen NK cells was further confirmed by RT-PCR (data not shown).

**Figure 3:**
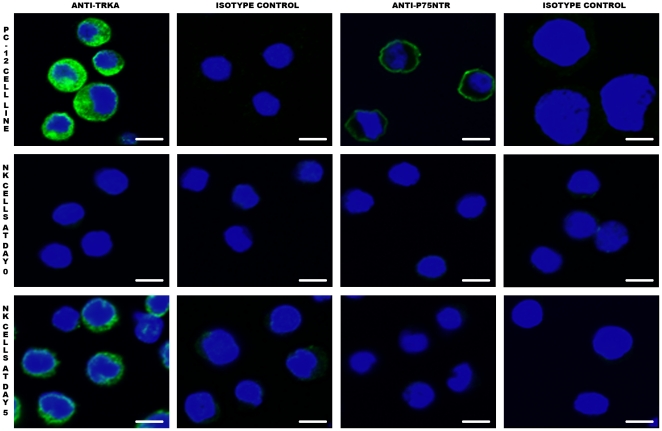
TrkA expression on NK cells as assessed by confocal microscopy. Cells were prepared for confocal microscopy as described in Materials and Methods. TrkA, but not p75^NTR^, is expressed by activated NK cells (green staining). On fresh NK cells (NK Day 0), the expression level of TrkA is probably too low to be evidenced by confocal microscopy, in contrast to flow cytometry. PC12 is a rat pheochromocytoma cell line that serves as positive control, as it expresses both TrkA and p75^NTR^ at high levels. The bar corresponds to 3 µm. Data shown are from one representative experiment out of three performed.

When spleen NK cells were activated by culture in the presence of IL-2, expression of TrkA increased gradually and progressively. At day 2, half of the NK cells were TrkA+, and then the percentage increased to nearly 100% until day 5 (Fig. 2A, Fig. 2C). This maximal percentage did not change until day 7 (Fig. 2A). Expression of p75^NTR^ was very moderately induced under these culture conditions (Fig. 1). Confocal microscopy confirmed the expression of TrkA by activated NK cells (Fig. 3). Despite the staining of a fraction of resting NK cells with the anti-TrkA Ab in flow cytometry, we could not detect TrkA-expressing fresh NK cells by confocal microscopy (Fig. 3). This suggests that the sensitivity of detection is higher in flow cytometry than in confocal microscopy.

**Figure 4:**
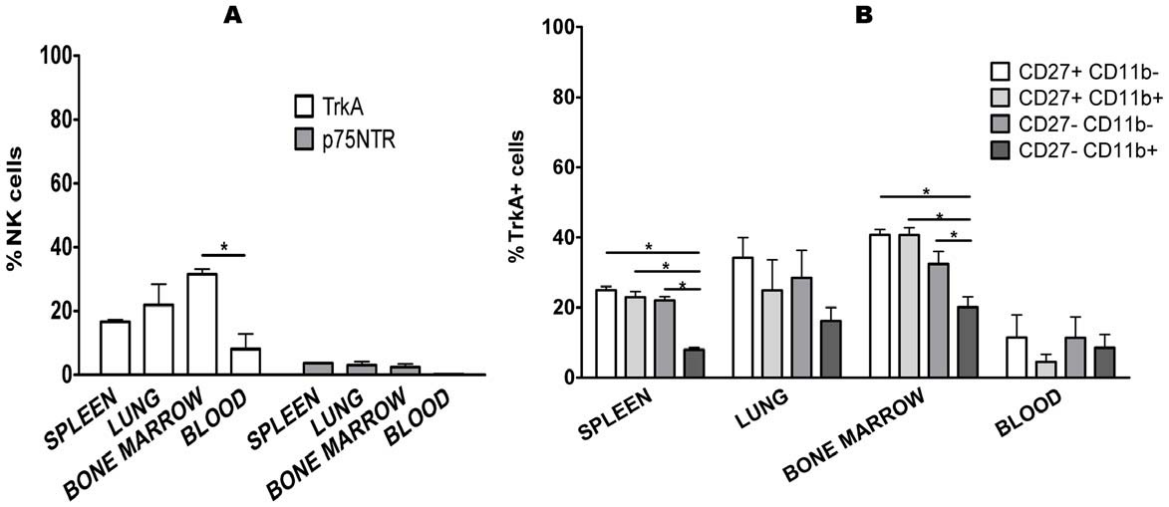
Expression of TrkA on NK cells is different depending on the organs and the NK cell subpopulation. A: NK cells (CD3-NK1.1+) from spleen, lung, bone marrow and peripheral blood were stained with anti-TrkA and anti-p75^NTR^ Ab and analyzed by flow cytometry. Dead cells were excluded by staining with Live Dead cell marker. The percentage of TrkA+ NK cells is highest in bone marrow and lowest in blood, whereas p75^NTR^ was expressed only on 3% of NK cells from spleen, lung and bone marrow. B: NK cells (CD3-NK1.1+) from the above mentioned organs were subdivided on the basis of the relative expression of CD27 and CD11b. TrkA expression is lowest on the CD27-CD11b+ population. The data shown are the means ± SEM of three experiments. *: p<0.05.

Then, we checked the expression of TrkA in fresh NK cells from mouse bone marrow, blood and lung. In lung, it was similar to spleen, in bone marrow, the percentage of TrkA-expressing cells was higher (approximately 30%) than in spleen, and in blood (approximately 15%) it was lower than in spleen (Fig. 4A). In contrast, p75^NTR^ was only present on 3% of the NK cells in all the organs tested, except in blood where it was absent (Fig. 4A).

Resting NK cells can be divided into different subsets and maturational stages according to the relative expression of CD11b and CD27 [6], [11], the CD27-CD11b+ population being considered as the most mature. In spleen, expression of TrkA was irregularly distributed, given that only 7.93% ±1.05 SEM of the CD27-CD11b+ NK cells were receptor-positive, while approximately 20% of the other three subsets expressed TrkA (Fig. 4B). The same distribution, at a lower percentage of TrkA+ NK cells in the CD27-CD11b+ population than in the other subsets, was observed in lung and bone marrow (Fig. 4B). It therefore seems that the percentage of TrkA+ NK cells is inversely correlated to the maturational stage of these cells.

Thus, we show here for the first time that a substantial fraction of fresh mouse NK cells expresses the NGF receptor TrkA, and that this fraction increases to 100% of IL2-activated NK cells.

### NGF does not influence NK cell proliferation

**Figure 5:**
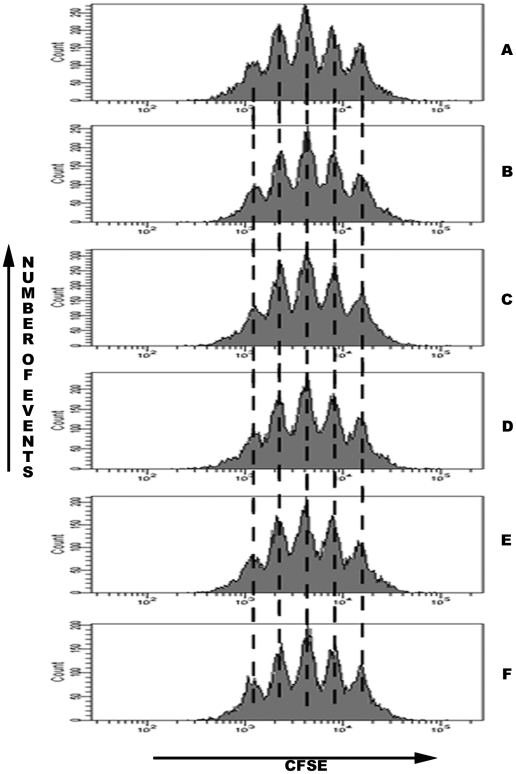
NGF does not influence NK cell proliferation. CFSE-stained splenocytes were cultured for three days in the presence of IL-2 and NGF at various concentrations; A: 0 pM (0 ng/ml), B: 0.1 pM (0.001 ng/ml), C: 1 pM, D: 3 pM, E: 10 pM (0.1 ng/ml), F: 10^4^ pM (100 ng/ml). Then NK cells (CD3-NK1.1+) were analysed by flow cytometry. Every peak of CFSE fluorescence corresponds to one generation of cells. Data shown are from one representative experiment out of three performed.

We evaluated NK cell proliferation after staining of the cells with the intracellular dye CFSE. NK cell division was evaluated after culture for three days in the presence of IL-2 and varying concentrations of NGF (from 0 to 10^4^ pm). Although cell proliferation was intense in all conditions, NGF had no influence on the proliferation rates, whatever the concentration (Fig. 5).

### Phenotype of NK cells activated in the presence of NGF

**Figure 6:**
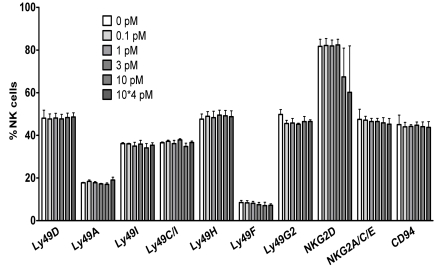
NGF does not modulate NK cell surface marker expression. Splenocytes were cultured during three days in the presence of IL-2 (1000 U/ml) and NGF at different concentrations. Then NK cells (CD3-NK1.1+) were analysed by flow cytometry for the expression of the surface markers indicated on the X-axis. Dead cells were excluded by staining with Live Dead cell marker. There were no significant differences between the two culture conditions for any of the markers tested (p>0.05). The data represent the mean ±SEM of three different experiments.

We also looked at the upregulation of the activation marker CD69, which was similarly high (expression by more than 80% of the NK cells after three days in culture with IL-2) in the presence or absence of NGF (data not shown). Likewise, no differences in the percentage of positive cells nor of the expression levels (reflected by mean fluorescence intensities) of various NK cell receptors (Ly49 family, NKG2A/C/E, CD94, NKG2D, adhesion molecules CD11a, CD18 and CD54) appeared after three days of culture with IL-2 alone or with IL-2+ NGF (Fig. 6). These results suggest that NGF does not influence the expression of surface molecules important for NK cell activation or inhibition.

### NGF does not induce apoptosis of IL-2-activated NK cells but does not promote survival

Apoptosis assays were performed by means of the annexin V/PI assay on NK cells cultured for three days in the presence of IL-2 alone or of IL-2+ NGF. The percentage of cells in early apoptosis (annexin V+PI-), in late apoptosis (annexin V+PI+) or already dead (annexin V-PI+) was not different between the two culture conditions (data not shown), suggesting that NGF does not induce apoptosis but also does not promote survival when the NK cells are activated with IL-2.

When cultured with NGF alone or with NGF + IL-2 at 100 U/ml (1/10 of the usual dose), no cells survived in the culture (data not shown), which could indicate that NGF is not a survival factor for NK cells.

### NGF partly inhibits NK cell degranulation but not IFNγ production

After triggering of their activating receptors, NK cells degranulate and release the cytolytic factors perforin and granzymes. This can be quantified through staining of CD107a, an intracellular granule marker that appears on the cell surface after degranulation [12], [13].

**Figure 7:**
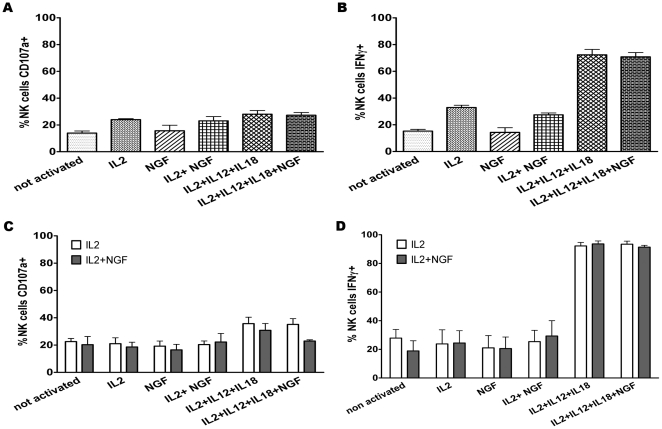
NGF reduces NK cell degranulation but does not influence IFNγ production. A and B: Fresh splenocytes were assessed for degranulation (A), reflected by the percentage of CD107a+ NK cells (CD3-NK1.1+), and for cytokine production (B), reflected by the percentage of IFNγ+ NK cells (CD3-NK1.1+). C and D: the same experiment was performed on NK cells (CD3-NK1.1+) cultured for three days in the presence of IL-2 alone (open histograms) or of IL-2+ NGF (100 ng/ml, filled histograms). The white bars correspond to cells cultured during three days with IL-2 alone, and the grey bars correspond to cells cultured with IL-2+ NGF during the same period. On day three, cells from both culture conditions were incubated during five hours in the different conditions shown on the X-axis. Dead cells were excluded by staining with Live Dead cell marker. NGF inhibits degranulation to a certain extent in the condition IL-2+ IL-12+ IL-18+ NGF. The data shown are the means ± SEM of three experiments.

At day 0, in freshly isolated NK cells, neither NGF nor IL-12+ IL-18 modulated the percentages of degranulating cells (Fig. 7A). NGF did also not influence the numbers of IFNγ-producing NK cells at day 0 (Fig. 7B).

After three days of culture in complete medium supplemented with IL-2 with or without NGF, NK cells were restimulated in different ways (cf Materials and Methods for details of the procedure). IL-12+ IL-18 increased the percentage of CD107a+ NK cells compared to the other conditions. In contrast, if NGF was added to these two cytokines, fewer NK cells degranulated. This difference did however not reach statistical significance (Fig. 7C).

When we measured IFNγ production in the same experiments (at day 3), no differences were noted between NK cells cultured in the presence of NGF compared to its absence (Fig. 7D).

In summary of this part, it appears that NGF has a modestly inhibiting effect on NK cell degranulation but it does not influence IFNγ production.

### Activated NK cells do not produce NGF

**Figure 8:**
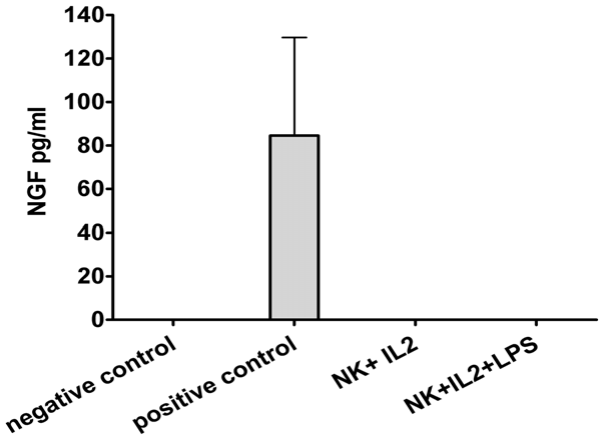
Activated NK cells do not produce NGF. Supernatants from purified NK cells activated for three days with IL-2 alone or with IL-2+ LPS are harvested and tested for NGF by ELISA. The negative control is culture medium (DMEM +10% FCS) and the positive control is BALF from a Balb/c mouse with allergic airway inflammation (induced by ovalbumin). The data shown are the means ± SEM of three experiments.

Purified NK cells were cultured during four days in the presence of IL-2 alone or IL-2+ lipopolysaccharide (LPS), known to induce NGF production in monocytes/macrophages [14]. Supernatants harvested from both NK cell culture conditions did not contain any NGF (Fig. 8), in contrast to the positive control. This control consisted in bronchoalveolar lavage fluid (BALF) of mice sensitized to ovalbumin in the context of another project. It is known that BALF of mice with an allergic inflammation of the airways contains high levels of NGF [15]. This showed that the ELISA assay efficiently detected NGF and that the failure to demonstrate NGF production by NK cells was not due to a technical problem.

## Discussion

In this paper, we show that around 20% of freshly isolated mouse NK cells express the high affinity NGF receptor TrkA. This expression is different on the various NK cell subpopulations defined by the presence of the surface markers CD27 and CD11b, the lowest percentage of TrkA+ NK cells being present within the most mature subset (CD27-CD11b+). Thus, TrkA might be a marker of relatively immature NK cells. Our observation that a higher percentage of NK cells from bone marrow as opposed to NK cells from spleen and blood express TrkA fits well with this concept. It would therefore be interesting to check the expression of TrkA on immature NK cell precursors in bone marrow. On the other hand, TrkA is strongly upregulated during NK cell activation, reaching 100% of the cells after 5 days of culture in the presence of IL-2. This is not contradictory to the low percentage of TrkA+CD27-CD11b+ NK cells, as the CD27-CD11b+ subset is actually in replicative senescence [6], [11] and therefore unlikely to contribute to the NK cell population obtained after culture.

Bracci-Laudiero et al. have shown that human umbilical cord blood CD34+ cells homogeneously express relatively high levels of TrkA and also produce NGF [16], which is the case to a much lower extent in cord blood mononuclear cells and adult PBMC (T cells, B cells, monocytes). These findings suggest that NGF could have its most important effects on hematopoietic precursor cells and/or during their differentiation towards mature cells of the immune system. NGF could then be one of the factors driving the development of mature peripheral NK cells from their precursors in bone marrow.

Likewise, NGF could be predominantly produced by immature cells, which could be the reason why we did not observe any NGF release in cultures of IL-2-activated splenic NK cells. However, other studies have described that activated, mature immune cells also can produce NGF: for example monocytes/macrophages, as already stated [14] or human CD4+ T cell clones [17]. It could be possible that IL-2 alone and IL-2+ LPS are not the appropriate molecules to induce NGF production by NK cells, or simply that NK cells are unable to produce and secrete NGF.

In contrast to TrkA, the pan neurotrophin receptor p75^NTR^ is not expressed by NK cells (neither resting nor activated). This is in accordance with the recent paper by Rogers et al. showing that resting human NK cells likewise do not express this receptor (whereas it appears intracellularly in the case of stimulation with IL-12) [4]. These authors also demonstrate the presence of TrkA on approximately 45% of human peripheral blood NK cells [4]. We could not confirm the data as we found, by using another Ab clone from the same supplier, only a very low percentage of TrkA+ human NK cells (data not shown). These discrepant results suggest that TrkA expression by human NK cells could be a donor-dependent phenomenon, or that they are due to the use of two different Ab. The clone used by Rogers et al. (165126) is sold as an Ab working in Western Blots, whereas the clone we used (165131) is recommended for flow cytometry. A third study investigated neurotrophin receptor expression in human PBMC and found, with yet a different Ab from a different supplier, that CD16+CD56+ NK cells express TrkA with a very low fluorescence intensity [18]. However, the authors did not gate out CD3+ T cells, so that they looked in fact at a mixture of NK cells and NKT like T cells.

As signaling through TrkA usually stimulates the cells and promotes survival [1]–[3], we performed functional assays which did not reveal any effect of NGF on NK cell survival, proliferation and cytokine production. In contrast, there is a tendency to a reduced activated NK cell degranulation in the presence of NGF. The lack of influence of NGF on most NK cell properties might come as a surprise, as the NGF receptor TrkA is not only present on NK cells but seems to be dynamically regulated. In accordance with our findings, it has been described that NGF has no influence on T cell proliferation and cytokine production [19]. On the other hand, there are also studies showing that NGF stimulates T cell proliferation [2], [3], and the differences might be related to the different experimental models.

In addition to its effect on degranulation, NGF might act as a chemotactic factor for NK cells. Indeed, this neurotrophin has been found to increase CXCL12-mediated attraction of monocytes and macrophages [20], [21] without modifying their antigen presentation capacity and the production of proinflammatory cytokines [20].

We ruled out the possibility of a biological inactivity of the NGF we purchased, as all the aliquots could induce neurite outgrowth in the pheochromocytoma cell line PC12 (data not shown).

It could be interesting to separate fresh TrkA+ from TrkA- NK cells and to repeat all of the above mentioned experiments. Likewise, NK cell development from bone marrow precursors could be investigated in the presence or absence of NGF, and the chemotaxis hypothesis could be tested. This however goes beyond the scope of the current manuscript and will be the focus of future research.
